# Explantation of Implantable Collamer Lenses due to high intraocular pressure in a highly hyperopic patient: a case report

**DOI:** 10.1186/s12886-026-04757-1

**Published:** 2026-03-24

**Authors:** Liuqing Cui, Karl Mercieca, Leonie Bourauel

**Affiliations:** Department of Ophthalmology, University of Bonn, Ernst-Abbe-Straße 2, 53127 Bonn, Germany

**Keywords:** Hyperopia, Implantable Collamer Lens, IOP elevation, Angle closure

## Abstract

**Background:**

Implantable Collamer Lens (ICL) implantation is an effective treatment for correcting high hyperopia. However, late anterior segment complications remain insufficiently characterized. The simultaneous occurrence of angle narrowing, pigment dispersion, and intraocular pressure (IOP) elevation nearly two decades after implantation of a non-central-port ICL is exceptionally rare. This case illustrates the diagnostic and surgical challenges of late-onset angle narrowing in hyperopic eyes and outlines strategies to minimize iris injury during ICL explantation.

**Case presentation:**

A 38-year-old woman presented with progressive bilateral elevation of IOP occurring 15 years after implantation of non-central-port ICLs for high hyperopia. Slit-lamp examination revealed shallow anterior chambers with patent peripheral iridotomies, narrow angles with partial peripheral anterior synechiae, and pronounced pigment deposition on the corneal endothelium, ICL surfaces, and trabecular meshwork. Sequential ICL explantation combined with cataract extraction and posterior chamber intraocular lens implantation was performed. During surgery on the left eye, mechanical iris trauma resulted in a sectoral iris defect causing monocular diplopia and photic disturbances. Despite undergoing iris repair, the patient’s photic symptoms did not improve. In the right eye, preoperative cycloplegia and intravenous mannitol deepened the anterior chamber and prevented iris injury. Postoperatively, both eyes developed a transient fibrin reaction that resolved with topical anti-inflammatory therapy. At the 6-month follow-up, IOP and visual acuity remained stable in both eyes, although mild glare sensitivity persisted.

**Conclusions:**

This case demonstrates that late-onset angle closure and pigment dispersion can occur after ICL implantation, particularly in highly hyperopic eyes with shallow anterior chambers. Lifelong monitoring of anterior chamber configuration and IOP is therefore essential in these patients. When IOP elevation secondary to angle closure develops, combined ICL explantation and cataract surgery can effectively restore aqueous outflow and visual function. Adequate preoperative deepening of the anterior chamber and avoidance of intraoperative iris manipulation are critical to minimize postoperative dysphotopsia and monocular diplopia.

## Background

The posterior chamber phakic intraocular lens (PC pIOL) implantation has been shown to be a safe and effective surgical procedure that provides stable and predictable refractive outcomes for patients with high hyperopia while preserving corneal anatomy [1]. However, there are a wide variety of long-term complications following Implantable Collamer Lens (ICL) implantation, and ICLs without a central aperture are more prone to complications such as high intraocular pressure (IOP), secondary glaucoma, and cataract [2]. We report the case of a patient with high hyperopia and astigmatism who developed bilateral IOP decompensation 15 years after ICL implantation.

## Case presentation

A 38-year-old woman with a family history of glaucoma was referred to our clinic due to elevated IOP in both eyes. She had undergone ICL implantation in both eyes in 2006 due to high hyperopia, (OD + 5.00D, OS + 5.75D). Upon presentation, IOP was 22 mmHg in both eyes under IOP-lowering treatment with bimatoprost, dorzolamide and clonidine.

According to the available medical records, the patient had been experiencing bilateral IOP elevations up to 30 mmHg without medication since 2021. The patient had been using bimatoprost since 2021, clonidine was added in 2023, and dorzolamide in spring 2024. Check-ups every three to four months repeatedly revealed IOP spikes and thus inadequate IOP control, which is why she was referred to our clinic in July 2024.

Best-corrected visual acuity (BCVA) was 20/20 in both eyes (OD -0.25/-2.0/19°) and OS -0.25/-3.0/167°). Slit-lamp examination revealed well-positioned PC pIOLs without central port, bilateral peripheral iridotomies at 1 o’clock and 11 o’clock were open. Gonioscopy showed partially occluded chamber angles, in OD nasal and superior Shaffer 0 with small peripheral anterior synechiae (PAS) at 1 o’clock, temporal and inferior Shaffer 3 (pigmentation level Scheie II-III), in OS superior Shaffer grade 0, nasal and inferior Shaffer 3–4 (pigmentation level Scheie III) and PAS from 5:30 to 0 o’clock. Visual field examination did not reveal characteristic glaucomatous visual field defects. High-resolution OCT examination displayed small optic discs in both eyes, with a CDR of 0.1 in OD and 0.15 in OS. As there was no evidence of glaucomatous damage, we agreed with the patient to adopt a wait-and-see approach and continue with medication.

**Fig. 1:**
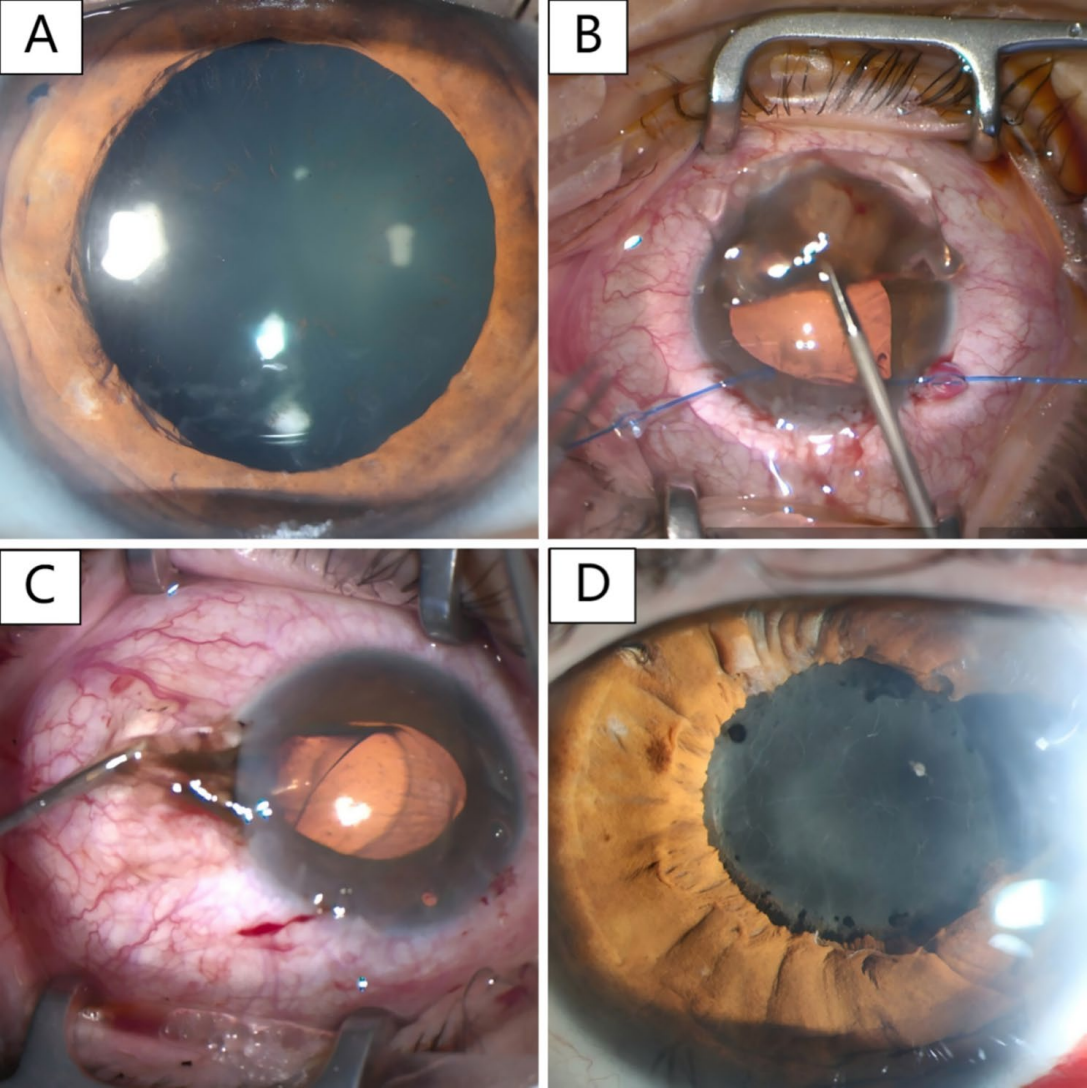
Photographs of OD and OS. (**A**) OD: Preoperative slit lamp examination - shallow anterior chamber, well-positioned ICL and peripheral iridotomies, posterior corneal and anterior ICL surfaces pigmented, and lens with anterior subcapsular opacity. (**B**) OD: A pupil dilator was placed in each of the two lateral incisions and the ICL was smoothly removed through the main incision. (**C**) OS: Passage of the ICL through the temporal clear corneal incision with forceps caused iris prolapse. (**D**) OS: Postoperative slit lamp examination at 12 days showed reticulofibrillar protein deposits in the anterior chamber, IOL in good position and temporal iris defects

Three months later, the patient was referred to us a second time with inadequate IOP control. IOP was 18 mmHg in OD and 22 mmHg in OS. Subcapsular opacities, increased pigmentation on the IOL, and partial adhesion between iris, IOL and lens were observed in both eyes (Fig. 1A). Due to borderline IOP in OS, explantation of the ICL was recommended. However, as the patient did not want to rely on high-prescription glasses after surgery and had never been able to tolerate contact lenses, we offered a combined explantation of the ICL and phacoemulsification with implantation of a posterior chamber IOL. Preoperative parameters are described in Table 1.

**Table 1:** Preoperative parameters captured via biometry and corneal pachymetry

Preoperative Parameters	OD	OS
Axial length (mm)	21.48	21.32
Anterior chamber depth (mm)	2.39	2.31
Horizontal white to white diameter (mm)	11.4	11.4
Anterior chamber volume (mm³)	79	52
Anterior chamber angle (180°)	15.3	11.8
Pupil diameter (mm)	3.79	3.61
Vault Height (µm)	560	670
Lens thickness (mm)	3.89	3.92
Central Corneal Thickness (µm)	545	550
Corneal Astigmatism (D)	-1.8	-2.7
K_max_ (D)	44.7	45.6

When removing the ICL from OS, adhesions between ICL and iris were released first, subsequent attempts to mobilise the ICL resulted in iris prolapse. After removal of the ICL, the iris could be repositioned and phacoemulsification with implantation of a foldable hydrophilic posterior chamber IOL (CT ASPHINA 409 MP 29.0D) was performed without complications. At the end of surgery, dexamethasone was injected subconjunctivally to reduce the expected postoperative inflammation. One day post-surgery, BCVA was 20/63 with an IOP of 18 mmHg. Slit-lamp examination revealed mild intraocular inflammatory response without fibrin. Postoperative treatment therefore consisted of dexamethasone eye drops 6 times daily and ofloxacin 4 times daily. The IOL was found to be positioned correctly, and iris defects were observed at 3 o’clock (Fig. 1C). One week post-surgery, BCVA in OS was 20/80, IOP was 18 mmHg. The patient reported decreased visual acuity since the previous day. Slit lamp examination revealed fibrin deposition in the anterior chamber (Fig. 1D). In addition to dexamethasone eye drops administered every thirty minutes, ofloxacin four times daily, scopolamine hydrobromide drops twice daily, and prednisolone pivalate administered at night, four subconjunctival injections of dexamethasone 4 mg were given. Over the course of the following month, local therapy could be reduced as intraocular inflammation decreased and BCVA increased to 20/20. IOP in OS at one month post surgery was 15 mmHg without medication, after three months 16 mmHg and after six months 14 mmHg (with dorzolamide).

OD was classified as being at high risk of acute and/or chronic angle closure. Moreover, the patient reported problems due to anisometropia, wanted to avoid wearing glasses if possible and could not tolerate contact lenses. It was furthermore assumed that earlier surgery on the second eye would reduce the risk of complications such as those that had occurred in the first eye. All advantages and disadvantages (including loss of accommodation) as well as intra- and postoperative risks (including iris trauma and postoperative inflammation) were discussed in detail with the patient, but as she found the permanent local therapy and the risk of developing glaucoma to be a great burden, we decided to procede with combined ICL explantation and cataract surgery in OD. Mannitol was administered intravenously half an hour prior to surgery. To prevent iris tearing, iris retractors were inserted, the ICL was then carefully removed (Fig. 1B). Subsequently, a gentle goniosynechiolysis to restore the anatomical conditions and phacoemulsification with implantation of a foldable hydrophilic posterior chamber lens (CT ASPHINA 409 MP 29.0D) were performed without complications. At the end of surgery, dexamethasone was injected subconjunctivally to minimize risk of postoperative inflammation and acetazolamide was given once. BCVA in OD was 20/40 one day post-surgery, IOP was 14 mmHg (with dorzolamide), the anterior chamber deep with minimal cells. OS had an IOP of 16 mmHg, and showed minimal fibrin deposits. Dexamethasone eye drops were administered six times daily.

One week post-surgery, BCVA was 20/16 in both eyes, IOP was 13 mmHg in OD. Slit lamp examination showed fibrin deposits in the anterior chamber. The patient received hourly prednisolone drops and bimatoprost, dorzolamide and atropine in OD. This led to a quick decrease in inflammation, so that both steroids and IOP-lowering therapy could be reduced and then discontinued. IOP remained stable with 17 mmHg one month after surgery (with dorzolamide), 14 mmHg after three months and 13 mmHg after six months.

**Fig. 2:**
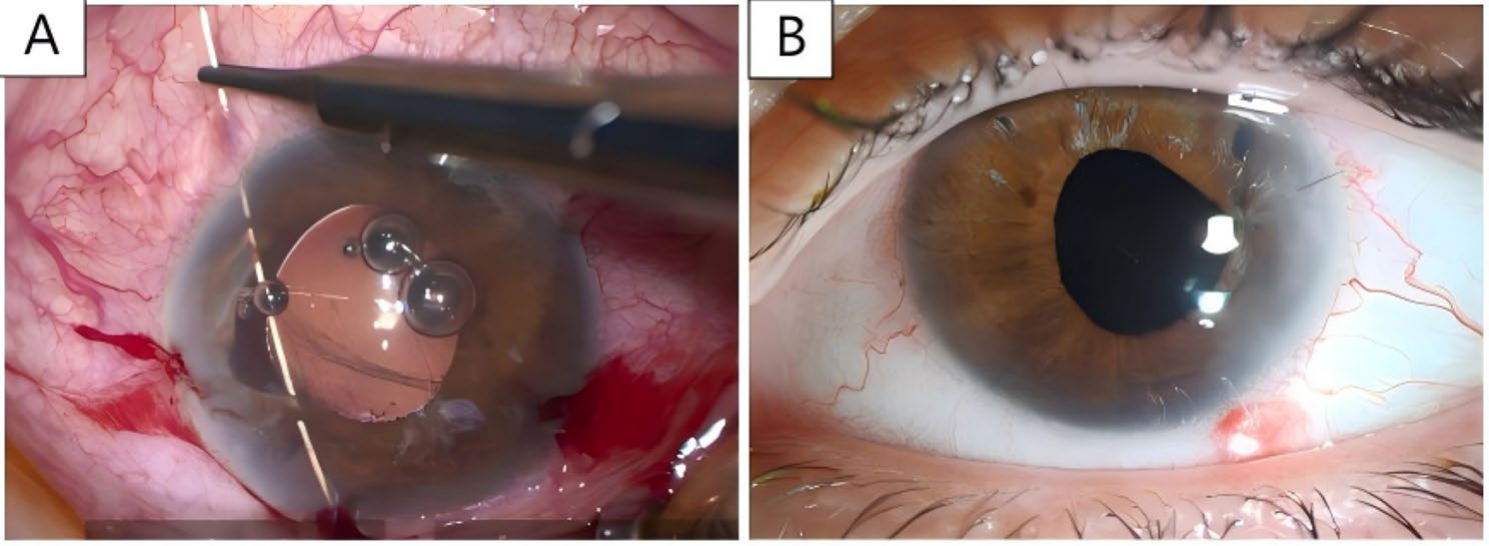
Photographs of OS. (**A**) Iris suturing: A single straight needle was used to pass through the 2 edges of the iris defect and was taken out of the anterior chamber through another limbal paracentesis before being cut. (**B**) First day post iris suturing surgery: The pupil remains irregularly shaped

However, the patient complained of persistent glare sensitivity in both eyes. For this reason, pilocarpine was applied in OS twice daily to determine whether a smaller pupil (i.e. pharmacological miosis) would help reduce glare sensitivity before planning surgical pupilloplasty which was subsequently performed under general anaesthesia without complications (Fig. 2). Both eyes underwent YAG laser capsulotomy for the treatment of posterior capsular opacification.

Six months post-surgery, the patient’s eyes remain highly sensitive to glare despite stable IOP. Pilocarpine drops were discontinued, and patient was advised to wear edge-filter glasses to reduce glare sensitivity.

## Discussion and conclusions

To the best of our knowledge, this case represents a long interval between ICL implantation and the development of chronic IOP elevation in a highly hyperopic eye [3–5]. Such an unusually delayed presentation indicates that progressive angle narrowing may arise many years after implantation and should remain a diagnostic consideration even in long-term follow-up.

ICL implantation is frequently associated with a number of potential complications, high IOP and secondary glaucoma account for 4.4% of the complications after ICL implantation [6]. Pigment dispersion has been identified as a potential cause of chronic IOP elevation [7]. Previous reports have described late-onset pigment dispersion or IOP elevation after ICL implantation, but most cases involved myopic eyes and occurred within a much shorter interval. These reports typically featured classic pigment dispersion with open angles and marked trabecular pigmentation [4, 5]. In contrast, the mechanism in our patient differed substantially. Chronic IOP elevation resulted primarily from progressive angle narrowing with PAS rather than a classic pigment-dispersion mechanism. Gonioscopy revealed partial angle closure with PAS in both eyes, and biometric data showed shallow anterior chamber depth (ACD), narrow anterior chamber angle (ACA, < 20°), and a relatively high vault. These anatomical factors, especially in a hyperopic eye with a short axial length and preexisting narrow angles, predispose to long-term angle closure and impaired aqueous outflow. Consistent with this, the patient’s IOP was more difficult to control in the left eye, which had a narrower angle and more extensive PAS. While pigment deposition was observed on the IOL and trabecular meshwork, the accumulation is more likely a secondary effect of angle closure and disrupted aqueous dynamics.

Highly hyperopic eyes present unique challenges in ICL implantation. Their smaller globe size, shallow ACD, and reduced ACA increase the risk of postoperative complications, including angle closure, anterior subcapsular cataract (ASC), and elevated IOP [8, 9]. The original non-central-hole ICL models are particularly prone to disturbing aqueous flow and pigment dispersion, necessitating peripheral iridotomy (PI). In our patient, angle narrowing and pigment accumulation developed despite a patent PI, highlighting the inherent vulnerability of hyperopic eyes to long-term angle compromise. Although modern ICL designs with a central port have reduced these risks, individual anatomic limitations remain critical. Vault height is another critical parameter, both insufficient (< 250 μm) and excessive vault (> 750 μm) are associated with complications such as ASC and angle closure, respectively [10]. In this case, the vault height fell within an intermediate range yet still contributed to angle crowding, likely reflecting the naturally constrained anterior segment of a highly hyperopic eye.

ASC is a well-recognized complication of ICL implantation, typically resulting from ICL-lens contact [2]. Combined ICL explantation and phacoemulsification with posterior chamber IOL implantation is an effective and safe management strategy, particularly in hyperopic patients [11]. In the present case, ICL removal resulted in IOP reduction, likely due to restoration of aqueous outflow through previously appositionally closed angles and possibly to the resolution of a pupillary block. Preoperative imaging and gonioscopy revealed narrow angles with partial angle closure and PAS, particularly in the left eye. Following ICL explantation and lens removal, the anterior chamber deepened, the iris-lens diaphragm shifted posteriorly, and the previously compressed angle structures reopened. This anatomical restoration significantly improved trabecular outflow and contributed to the normalization of IOP.

Mechanical iris injury occurred during the separation of PAS in the left eye and during ICL removal, whereas no intraoperative complications were noted in the right eye after preoperative intravenous mannitol administration and the use of a pupil dilator. Iatrogenic iris damage to the iris can result in an irregularly shaped pupil, causing symptoms such as photophobia, glare and diplopia in patients, and iris suturing may help alleviate these symptoms [12]. A pupilloplasty was therefore performed on the left eye, but the procedure did not lead to meaningful improvement in glare sensitivity. Due to the severe iris damage and the pupil’s inability to return to a regular circular shape, further iris suturing would have impacted the patient’s scotopic vision. Therefore, a conservative treatment plan was adopted. This experience illustrates the importance of preventing such complications. Consequently, perioperative control of IOP and appropriate intraoperative protection of the iris may reduce pupil-related visual problems after PC pIOL implantation in highly hyperopic eyes.

In highly hyperopic patients - especially those with a family history of glaucoma - ICL implantation requires careful anterior segment assessment, thoughtful lens selection, and close long-term IOP and angle monitoring. Central-port designs reduce risks such as pupillary block and pigment dispersion, but eyes predisposed to angle closure or requiring PI need heightened surveillance. Refractory IOP elevation demands mechanism-based management and may necessitate explantation, which can restore IOP control. However, the shallow anterior chamber in hyperopic eyes increases surgical difficulty and risk of iris trauma, emphasizing meticulous technique and careful patient selection.

## Data Availability

All relevant data supporting the findings of this study are included in the manuscript.

## Abbreviations

PC PIOL: Posterior Chamber Phakic Intraocular Lens
ICL: Implantable Collamer Lens
IOP: Intraocular Pressure
BCVA: Best-Corrected Visual Acuity
PAS: Peripheral Anterior Synechiae
KP: Keratic Precipitates
ACD: Anterior Chamber Depth
ACA: Anterior Chamber Angle
ASC: Anterior Subcapsular Cataract
PI: Peripheral Iridotomy
